# A study measuring the degree of integration between the digital economy and logistics industry in China

**DOI:** 10.1371/journal.pone.0274006

**Published:** 2022-09-22

**Authors:** Wei Zhang, Hongli Liu, Yuan Yao, Zifu Fan

**Affiliations:** School of Economics and Management, Chongqing University of Posts and Telecommunications, Chongqing, China; Szechenyi Istvan University: Szechenyi Istvan Egyetem, HUNGARY

## Abstract

Under the new development pattern, the penetration and integration between industries is becoming increasingly active, constantly promoting the rationalisation and heightening of the entire industrial structure and structuring new convergent industrial bodies. To explore the degree of integration between the digital economy and the logistics industry, this paper uses input-output models and social network analysis to empirically analyse the level of industrial integration between the core industries of China’s digital economy and the logistics industry during the period 2007–2017. The level of integration between the core industries of China’s digital economy and the logistics industry is measured, and its spatial and temporal characteristics are also measured and analysed. The analysis showed that (1) the degree of integration between the two industries is low, but with changes over time, the degree of integration will follow an upwards trend; the overall level of integration is increasing with the development of the core industries of the digital economy; the growth rate is significant, and developed regions have a clear advantage because of the intensity of integration. (2) The direction of industrial integration linkages is basically stable, with the pointing characteristics of adjacent provinces, and the spatial distribution shows a pattern of diffusion from the central region to the eastern and western regions. Overall, the integration shows an economic radiation effect, but there are large differences between the north and south. (3) It is easier for adjacent provinces to form the same cohesive subgroup in a network and develop together. At the same time, the integration network shows a clear pattern of marginal-semimmarginalization. The study enriches and improves the research status of China’s digital economy industry convergence, and provides theoretical support for the formulation of policies related to the synergistic development of China’s digital economy and logistics industry.

## Introduction

The digital economy, as a new economic form leading future economic development, is unprecedentedly driving changes in economic models and reconstructing economic development. It is an important force driving changes in the quality, efficiency and power of social and economic development. The digital economy has had a tremendous impact on the world economy, transcending physical or geographical limitations. The four fundamental technology areas closely related to the digital economy are the Internet of Things, cloud computing, big data analytics and artificial intelligence. Advanced digital technologies can accelerate the ongoing dynamics of organisation, job content and industry location choices in terms of mainly the subdivision of relevant economic activities into more professional business functions, the organisational and spatial split of low-skilled jobs from knowledge-intensive jobs and the automation of low-skilled jobs [1]. The global digital economy is dominated by developed economies [2] and countries are fully committed to promoting their own digital economies, which has intensified competition between countries. With the invention and diffusion of new digital technologies, competition between industries resulting from changes in the digital economy has become extremely intense and complex.

In the 19th National Congress, General Secretary Xi Jinping proposed the deep integration of the internet, big data, artificial intelligence and the real economy, and the 14th Five-Year Plan for National Economic and Social Development further proposed vigorously developing the digital economy and promoting digital industrialisation, industrial digitisation, and the deep integration of the digital economy and the real economy. According to “White Paper on China’s Digital Economy Development (2021)” released by the China Academy of Information and Communications Technology (CAICT), as a new economic form, the digital economy, with digital technology as the core driver, promotes the digital transformation and high-quality development of the global economy through three paths: new technology forms new industries, new industries give rise to new models, and new technology empowers traditional industries. The integration, transformation and reshaping of traditional industries through the digital economy has become an important means of developing new economic forms. Innovations brought about by digital technology will transform production, consumption, investment and foreign trade, and the use of automation and high-tech equipment will continue to increase in People’s Daily work and life. The emergence of service modes such as instant delivery and shared travel has led to great changes in the field of transportation and logistics.

**Fig 1:**
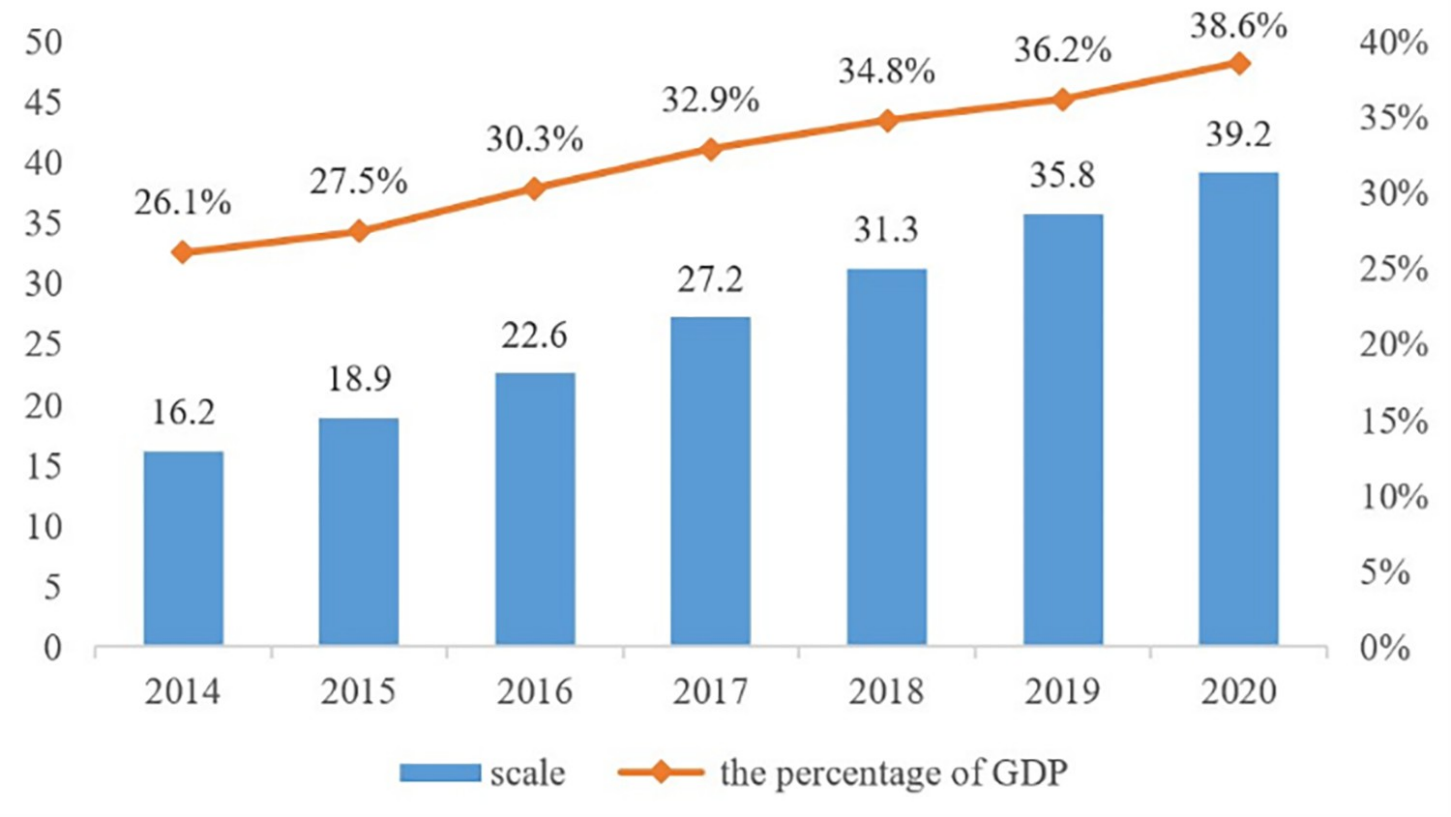
Scale of China’s digital economy and proportion of GDP.

The concept of China’s digital economy was first introduced at the G20 Summit in Hangzhou in 2016. After several years of development, the digital economy industry has made great progress. According to “the Digital China Development Report (2020)” released to the public by the State Internet Information Office in July 2021, China’s total digital economy has jumped to the second place in the world. the scale of China’s digital economy has reached 39.2 trillion yuan in 2020, and the added value of the core industries of the digital economy reached 7.8% of GDP (Fig 1). Although researchers still have different definitions of the connotation of the digital economy, existing studies generally agree that the digitalisation of industries can adjust the industrial structure, promote industrial transformation and upgrading, and improve productivity.

**Fig 2:**
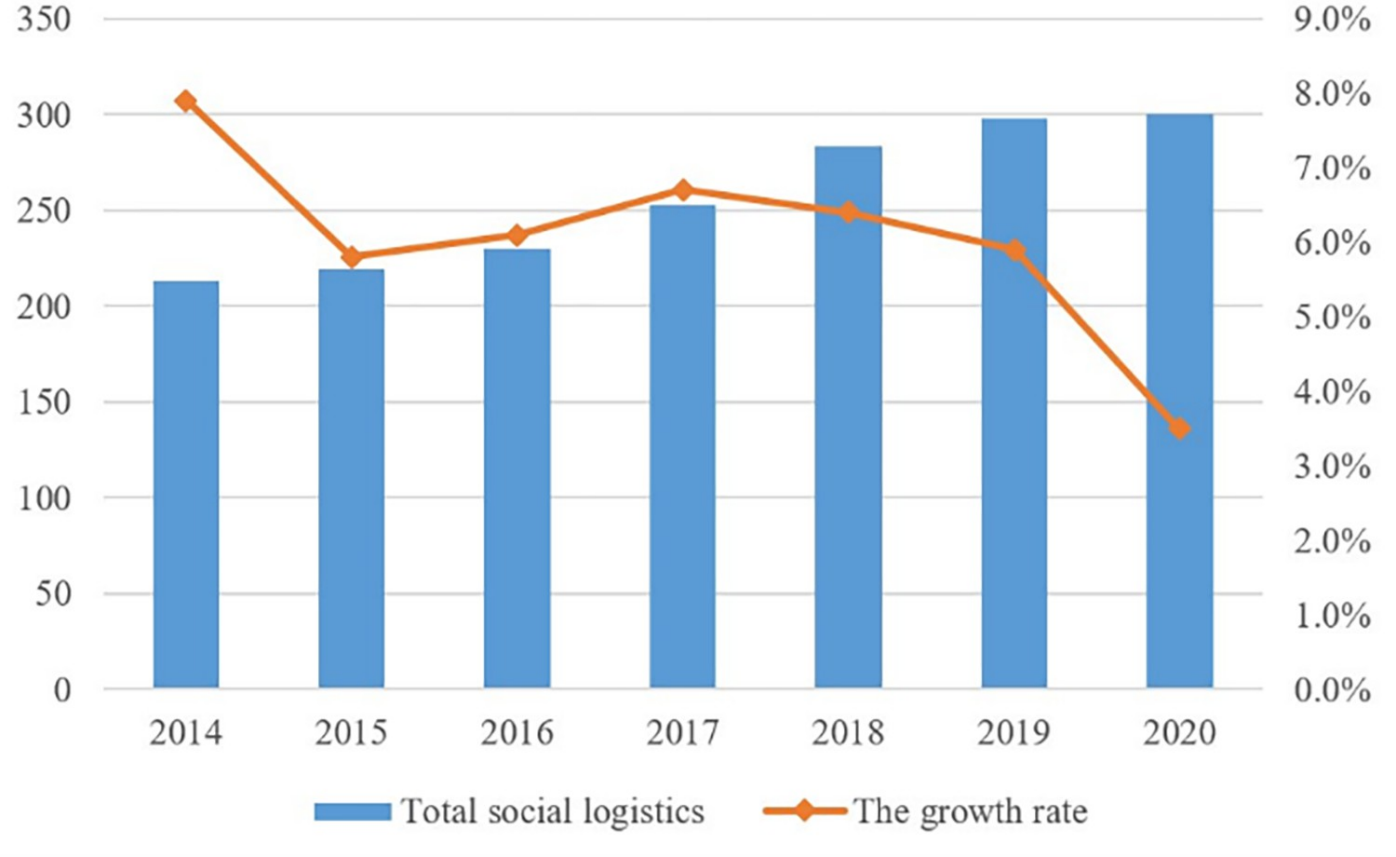
Trend of China’s total social logistics.

As an important practice subject of national economic development, the significance of the economic development of the logistics industry in driving China’s overall development and construction is self-evident. The 2006 logistics industry was written into the five-year plan for the first time, and under the policy encouragement of all parties, it began to actively explore the implementation of logistics modernization and development. The scale of China’s 2013 logistics market marked the first time since overtaking the United States to be ranked first in the world, 2014–2020 China’s social logistics maintained high growth (Fig 2); in 2018, total national social logistics equalled 283.1 trillion yuan, an increase of 6.4% year-on-year. In 2020, due to the coronavirus epidemic, China’s logistics industry suffered a large impact. With the prevention and control of the epidemic achieving significant results, logistics operations continued to restore momentum. In 2020, China’s total social logistics were 300.1 trillion yuan, an increase of 2.1 trillion yuan compared to 2019. China’s total social logistics in the first half of 2021 equalled 150.9 trillion yuan, and the figure for the entire year is expected to exceed that of 2020. The rapid growth of the total amount of logistics shows that the value creation ability of logistics industry is continuously enhanced, and the logistics demand of economic and social development and People’s Daily production and life is increasing, which effectively promotes the sustainable and healthy development of China’s national economy.

In the process of integrating the digital economy and the real economy, the logistics industry is an important pioneering industry and a bridge between the real economy and the digital economy. The initiative of the digital economy provides a rare opportunity for the rapid development of logistics modernisation in China. The digital economy is inherently highly integrated, and digital technology has a guiding role and a very high permeability function for the modernisation of all industries, reinforcing the trend of mutual integration of various industries through the link of information. The logistics industry and various industries are already naturally and inextricably linked, and this is reinforced by the role of digital ties. With the increasing application of a series of cutting-edge digital technologies such as cloud computing, big data, Internet of Things and artificial intelligence in the logistics industry, it has driven the digital transformation of logistics enterprises to accelerate, helping them to allocate resources scientifically and rationally, actively expand double-ended retail, open up the upstream and downstream of the supply chain, and derive new models and new business modes such as third-party and fourth-party logistics, which not only broaden the service areas of the logistics industry, but also improve and enhance This not only broadens the service area of the logistics industry, but also improves the experience of customers. At the same time, the integration of logistics and e-commerce has become increasingly close, effectively driving the demand for logistics services and revolutionising the logistics industry model. The cross-border development of the logistics industry with other industries has become a trend, digital collaboration across regions, systems and businesses has become the norm, and industrial governance capacity and governance systems have been strengthened, indicating that the logistics industry has started to enter the era of digital economy in a comprehensive manner.

The digital economy has provided a new impetus for the sustainable development of the logistics industry [3]. With the flourishing of the digital economy, a new generation of digital technologies has gradually been integrated into the production process of the logistics industry, and intelligent digital logistics as a new form of industry integration has emerged at the right moment. According to the "2021–2027 China Smart Logistics Industry Supply and demand Situation Analysis and Market Operation Potential Report" released by Zhiyan Consulting, the size of China’s smart logistics market has reached 500 billion yuan in 2019, with a year-on-year growth of 23.1%; In 2020, the scale of China’s intelligent logistics market will reach nearly 600 billion yuan, and is expected to exceed one trillion yuan by 2025. Under the background of the integration of digital economy and real economy, the logistics industry, as an important part of the real economy, has guiding significance for the transformation and upgrading of China’s logistics industry by promoting digitalization, intelligent transformation and cross-border integration, developing new circulation technologies, new business forms and new models, and opening up the era of "digital logistics"

How to deeply grasp the pulse of the development of digital economy, conform to the development law of digital economy and logistics industry, and explore the interactive development of digital economy and logistics industry from the theoretical and empirical research level is becoming the focus of attention and research of industry experts and scholars and entrepreneurs. Scholars are committed to systematically studying digital transformation and industrial structure, industrial layout, industrial coordination and other directions, comprehensively measuring the interaction between the core industry of digital economy and the development of logistics industry, and forming a theoretical paradigm for the integration of digital economy and traditional industry. This is of great significance to digital technology enabling industrial structure transformation, forming new economic growth points and promoting high-quality economic development. However, the existing literature on logistics digitalization is still insufficient. Currently, scholars generally focus on policy suggestions and theoretical research, and there are few empirical studies on the level measurement of the integration of logistics industry and digital economy. Based on this, this study attempts to review the existing literature on industrial integration measurement methods, combine the principles of rationality and availability of data, use the input-output method to study the integration development of the digital economy and logistics industry from a quantitative perspective in recent years, and integrate social network analysis to study the spatial and temporal regional characteristics, in order to provide a basis for measuring the industrial integration of logistics and digital economy in China.

## Literature review

The application of digital technologies such as Internet, block chain and big data has strengthened the network effect between industries and broken the boundary of traditional industrial connotation. The emergence of this new industrial model makes the integration of digital economy and real economy widely and continuously concerned by international institutions and relevant research institutions, policy makers and economic researchers in major countries. Although the integration of the digital economy with the real economy has received extensive and sustained attention from international institutions and major national research bodies, policy-makers and researchers specialising in economics. Studies on the digitization of industries have focused on agriculture and manufacturing with some success, while research on the logistics industry is just beginning. Some studies have demonstrated the driving effect of the digital economy on the logistics industry. Zhang et al. [4] explored the local transformation of China’s logistics industry from the perspective of the digital economy by analysing the relationship between the logistics industry and the digital economy. Lu [5] empirically proved that the development of China’s digital economy has produced a significant promotional effect on the improvement of logistics efficiency. However, there are still problems with the existing research that cannot be ignored. First, existing research neglects any measure of digitalization, while research on the accounting of the scale of the digital economy industry has just started. Second, there is a lack of quantitative research related to the digitalization of logistics, and existing research focuses mainly on the digitalization of logistics, conducting qualitative research and providing relevant development suggestions; so far, no study has examined industrial integration, including its measurement.

As an economic phenomenon, industrial convergence is developing vigorously in the world, which is of great significance to the high-quality development of economy. In particular, China has certain advantages in the field of digital economy. If the digital economy comprehensively serves the development of the real economy, the deep integration of digital economy and real economy can greatly promote the upgrading and transformation of the industrial structure of China’s real economy. At present, there are abundant researches on industrial convergence, mainly focusing on concept definition, convergence mode, convergence measurement and so on. From the perspective of concept definition, Marshall first proposed the idea of industrial integration. In his theory of division of labor, he clearly pointed out that with the continuous refinement of division of labor, the boundary between industries will gradually blur [6], and industrial integration is an inevitable outcome when the economy develops to a certain level. In existing research on industrial integration, many scholars have conducted theoretical analysis and empirical research on the motivation of industrial integration, and the effective evaluation and measurement of the degree of integration between industries has also become a key concern for scholars at home and abroad. At present, research on measuring the degree of industrial integration has not yet resulted in a unified standard and method, nor has it formed a comprehensive indicator to reflect the overall process of industrial integration [7] and thus cannot explain the panorama of industrial integration in a country or region [8]. Correlation measures currently measure industrial synergies between cities or countries, as well as the degree of interindustry linkages. In general, depending on the perspective and data used, researchers have used four main approaches to measure the degree of industrial integration.

First, the overall correlation between industries is used to measure integration. This approach uses mainly methods such as correlation coefficient and Herfindahl index to reflect the integration between industries by analysing the similarity or correlation between industries using data such as industry size or value added. For example, Fai et al. [9] used interindustry patent correlation coefficients to measure the degree of technological integration among four industry sectors in the US: chemical, electronics, machinery and transportation. Gambardella [10] and others used the Herfindahl index to measure the degree of integration of different businesses in the electronic information industry. However, such methods are more often used to determine the existence of industry integration trends. They neither reflect the degree of industry integration and the integration process well nor explain how industries are connected to each other [8]. The Herfindahl index and the interindustry patent correlation coefficient are simple to compute, but industry-level patent data are difficult to obtain.

Second, the statistical modeling method is used to measure the level of industrial integration, which mainly includes methods such as coupled coordination degree model, grey correlation model, econometric model, construction of indicator system, and linear regression analysis. Duysters and Hagedoorn [11] used linear regression analysis, Du [12] used regression analysis to explore the role of information service industry on the transformation and upgrading of manufacturing industry from the perspective of industrial integration. Chen et al. [13] Quantitative evaluation of the development of agricultural industry integration in Shanghai using entropy value method and grey correlation analysis. Fang et al. [14], Li et al. [15] established an evaluation index system for the integration development of the cultural tourism industry using a coupled coordination degree model. However, the design of the indicators is too subjective and does not reveal the structural dynamics in the process of industrial integration.

Third, the input–output method is used to measure the degree of integration between different industries. The input–output model was first proposed by the American economist Leontief [16] and is one of the main models for studying industrial linkages. This approach uses input–output table data between industries to reflect industrial integration by analysing the degree of strong input–output linkages between industrial sectors. Tarnamura [17] used the industry linkage analysis method to study the changes in industrial structure in Asia-Pacific countries, using the electronic communication industry sector and the transportation equipment industry sector as examples. Boschma et al. [18] applied the industrial association method to analyse the industrial sectors of 103 provinces in Italy from 1995 to 2003. They found that foreign trade is an important factor affecting changes in regional industrial structure. Li et al. [19] used the input–output method to estimate the degree of integration of Zhejiang and the national information industry with various manufacturing industries. The holistic nature of the input–output method fully reflects the internal linkages between industries.

Fourth, social network analysis (SNA) is used to analyse the integration relationship between industries. Compbell [20] was the first to introduce SNA into the analysis of industrial linkages and explored industrial linkages in the Washington region through a network diagram constructed from the input–output matrix between industrial sectors. As SNA can visualise industrial linkages, it can intuitively and easily reveal the intrinsic degree of association and dependence between industries. Li et al. [21] performed a targeted study on the innovation output of various provinces, cities and autonomous regions in China and analysed the structure of the spatially linked network of innovation output and the changes in its characteristics by combining the gravity model with the SNA method. Zhou et al. [22] and other researchers analysed the characteristics of the industrial spatial association network structure and its innovation drivers in major cities in the Yangtze River Economic Belt. These studies show that the SNA method has unique advantages in industrial spatial association research and can offer greater detail on structural information.

Each of the four methods mentioned above has its own strengths and weaknesses, so to further study the development of inter-industry integration, scholar-researchers have combined and innovated on the traditional methods. The study found that multiple methods were used in combination to explore the linkages between industries, avoiding the limitations of a single method. Zhang et al. [23] Constructed an index system for measuring the level of urban-rural industrial integration based on the DPSR model, used the longitudinal and horizontal pull-out grade method to comprehensively assess the level of urban-rural industrial integration, and combined kernel density estimation, hierarchical cluster analysis and spatial autocorrelation analysis to analyse and explore its spatio-temporal evolution characteristics. Peng [24] combined the coupled evaluation method with the DEA-Malmquist method to measure the level of industrial integration, and explored the positive impact of industrial integration on urban total factor productivity after introducing the spatial panel Durbin model for spatio-temporal analysis. These studies demonstrate the combination of multiple methods for measuring industrial integration, reducing the limitations of a single method and improving the accuracy of the measurement.

Some scholars such as Du et al. [25], Sun et al. [26] attempted to introduce SNA into the study of industrial association, used the input-output relationship data between industries to construct the relationship network between industries, and analyzed the changes of industrial roles and network structure in the network. Yang [27] attempted to use the input–output approach directly under the SNA paradigm to analyse the contagion process of economic shocks caused by changes in demand or supply in each industrial sector in the input–output network. These studies confirm that combining the two approaches can avoid the three major shortcomings of traditional methods of identifying and measuring industrial integration. This combination can effectively determine the existence and degree of industrial integration through changes in individual roles and network structure, thus taking the study of interindustrial linkages a step further. In addition to constructing social networks based on national-level input–output tables, more scholars are constructing the structural evolution of regional industrial networks based on regional input–output tables and SNAs. Sun [28] compared the isomorphic and heterogeneous characteristics of industrial linkages in Beijing, Tianjin and Hebei using complex network analysis based on regional input–output tables in 2012. In addition to analysing the industrial linkages of all industrial sectors in the country and the local region, some scholars have started to focus on the industrial linkages and internal economic network structures between industrial sectors. Based on input–output tables and SNA, Zeng [29] conducted an empirical study of the industrial linkages among service industry segments and between them and the manufacturing sector, while Zhu et al. [30] analysed the internal economic network structure of China’s service industry and its evolution. These studies confirm that the combination of the two can not only avoid the defects of the traditional identification and measurement methods of industry convergence, but also effectively judge the existence and degree of industry convergence through the changes of individual roles and network structure, which makes the research on inter-industry connection further. Therefore, the combination of input-output table and SNA in industrial association analysis is favored by industrial economics researchers at home and abroad.

The input-output method mainly measures the industrial integration formed by industrial penetration. Compared with the patent data used by correlation coefficient analysis method and Herfindahl index method, the input-output method is more suitable for both the data availability and the relationship between the measured industries. At the same time, both the digital economy and the logistics industry, which are the subject of this study, both have ambiguous boundaries and do not have physical boundaries, making it difficult to clearly explore their levels of industrial integration using index methods and econometric models. Compared with the existing literature, this paper measures the development of the integration of the logistics industry and the core industries of the digital economy in China based on the importance and inevitability of the integration of the logistics industry with the core industries of the digital economy. It uses input–output analysis and SNA to gauge the development of the integration of the logistics industry with the core industries of the digital economy and to provide a reference for policy formulation to promote the digital transformation of the logistics industry. Therefore, the contribution of this paper mainly lies in: First, it extends the study of industrial integration to the logistics industry and the core industries of the digital economy by measuring the level of integration between the logistics industry and the core industries of the digital economy in China and 29 provinces, cities and autonomous regions, focusing on the degree and status of industrial integration and its dynamic changes among provinces and cities, enrich the theoretical research in the field of industrial convergence. Second, it explores the factors influencing the integration of the logistics industry with core industries, provides empirical evidence of industrial integration, and establishes an empirical basis for improving the digitalisation level of China’s logistics industry. Third, it focuses on the temporal evolution and spatial correlation of the integration of logistics and the digital economic. The objective is to provide policy suggestions to guarantee the high-quality development of the logistics industry, narrow the regional digital economy gap, and promote coordinated regional development.

## Methodology

### Research methods

Input-output method, proposed by Vasily Leonseff, an American economist, is a quantitative method to study industrial association. Input-output table provides a concise and systematic structure model for the whole economic activity of a country or region. It studies and analyzes the quantitative dependence relationship between production and consumption among various sectors of the national economy from two directions of production consumption and distribution and use. Each production department needs to purchase products and pay service fees from other production departments, and also produce products and provide services to other departments. This quantitative dependence of input and output provides a starting point for the study of economic relations between industries. Therefore, this method is often used to analyze the correlation between industries and the impact of industries on the national economy, and it is a relatively mature tool to analyze the correlation between industries.

### Data sources

This paper draws on data related to logistics and the core industries of the digital economy. According to the industrial classification standard of the national economy formulated by the National Bureau of Statistics, the logistics industry belongs to the transportation, storage and postal industry in the tertiary industry, so the relevant data of transportation, storage and postal industry are used to calculate measures related to the logistics industry. The core industry of the digital economy is a new concept proposed by the National Bureau of Statistics in 2021 based on the classification statistics requirements of digital economy industries, including digital product manufacturing, digital product service, digital technology application and digital factor driven industries. The National Bureau of Statistics has not yet released relevant data on the core industries of the digital economy and has not developed ready-made statistical data. Based on existing research on the industrial division of the digital economy, combined with the digital economy core industry classification standard issued by the National Bureau of Statistics in 2021, the economic indicators of each core industry in the standard are used to represent the development level of the digital economy industry. According to “the Statistical Classification of Digital Economy and Its Core Industries (2021)” and “Industry Classification of National Economy (GB/T 4754–2017)”, the core industry scale of the digital economy can be estimated using the manufacturing of computers, communication and other electronic equipment. Information transmission and software and information technology services are the two core industry sectors to quantify. These two core industry sectors and the digital economy core industries are closely linked and numbers can to a great extent represent the core industry economic scale, growth rate and internal structure. Moreover, in terms of data availability and international comparability, they have obvious advantages; therefore, they are ideal economic indicators of core industry estimates.

Through the CEINET、Development Information Research Center of The State Council and National Bureau of Statistics website, which provides data on 31 provinces and autonomous regions in China in the form of resources such as the statistical yearbook, statistical bulletin, and city yearbook, can be accessed. These resources also provide information on the digital economy foundation industry of 31 provinces and cities in China and the logistics industry for the period 2007–2017, thus enabling the measurement of the national and provincial and municipal level of industrial convergence.

### Input–output analysis

By analysing the forwards and backwards correlation between the logistics industry and the core industry of the digital economy, the degree of correlation between the logistics industry and the core industry of the digital economy can be accurately and objectively reflected to discern the degree of integration between the two.

Since the input–output table describes the input of other sectors as intermediate goods in the production process of a particular sector, this paper will use the input–output analysis method to study the correlation between China’s logistics industry and the core industry of the digital economy.

The direct consumption coefficient is used to measure the direct backwards correlation between the logistics industry and the core industry of the digital economy in its production and operation. The higher the direct consumption coefficient is, the more products that are consumed by a certain department in the logistics production process, and the more dependent the latter is on the product or service input of the department. The formula is as follows:

$$A_{ij}=\frac{x_{ij}}{X_{j}}$$ (1)

where *x*_*ij*_ is the value of products or services directly consumed in the production process of the logistics industry from sector *i* in region *j*, the core industry of the digital economy, and *X*_*j*_ is the total output of the logistics industry.

The direct distribution coefficient is used to measure the degree of direct forwards correlation between the logistics industry and the industries that use its products as inputs or means of production. The larger the direct distribution coefficient is, the greater the direct demand for logistics services of the core industries of the digital economy is. The formula is as follows:

$$B_{ij}=\frac{y_{ij}}{X_{j}}$$ (2)

where *y*_*ij*_ is the value of the products or services provided by logistics industry *j* for intermediate use in sector *i* of the core industry of the digital economy.

#### Measure of the national integration level based on the input–output method

Overall, the investment rate and demand rate between the two industries are relatively low. In comparison, from 2007 to 2017 (Table 1), the investment rate of the core industry of the digital economy in the logistics industry is higher than that of the logistics industry in the core industry of the digital economy, indicating that the core industry of the digital economy is less dependent on the logistics industry than vice versa. The demand rate of the logistics industry for the core industry of the digital economy is higher than vice versa, indicating that relatively speaking, the core industry of the digital economy is more dependent on the consumption of the logistics industry, while the latter is less dependent on the consumption of the former. The above results show that the integration of the core industry of the digital economy and the logistics industry in China is not ideal, the logistics industry is not developed enough to support the development of the core industry of the digital economy, and the core industry of the digital economy is not developed enough to actively integrate with the logistics industry.

**Table 1:** Integration degree of the core industry and logistics industry in China’s digital economy.

Year	The output and input of the logistics industry to the core industry of the digital economy		The output and input of the digital economy core industry to the logistics industry		degrees of fusion: F
	Direct consumption coefficient: R1	Direct distribution coefficient: Q1	Direct consumption coefficient: R2	Direct distribution coefficient: Q2	
2007	0.027509	0.013598	0.007779079	0.015737	0.016156
2010	0.034087	0.010519	0.005594011	0.018127	0.017082
2012	0.03572	0.012301	0.006495344	0.018861	0.018344
2015	0.03781	0.013747	0.00693006	0.019060	0.019387
2017	0.044243	0.023944	0.011847292	0.021891	0.025481

**Fig 3:**
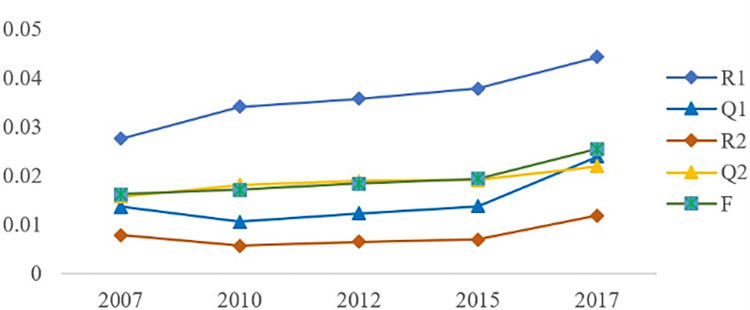
The integration level of China’s logistics industry and the core industry of the digital economy.

In Fig 3, although it can be seen numerically that the degree of integration between the two industries is low, from the perspective of time variation, the degree of correlation and integration of the two industries shows an upward trend. Overall, although the integration of the two industries is low, with the development of the core industry of the digital economy, their integration and ability of the two industries to offer mutual support are rising.

#### Measure of the provincial city integration level based on the input–output method

**Fig 4:**
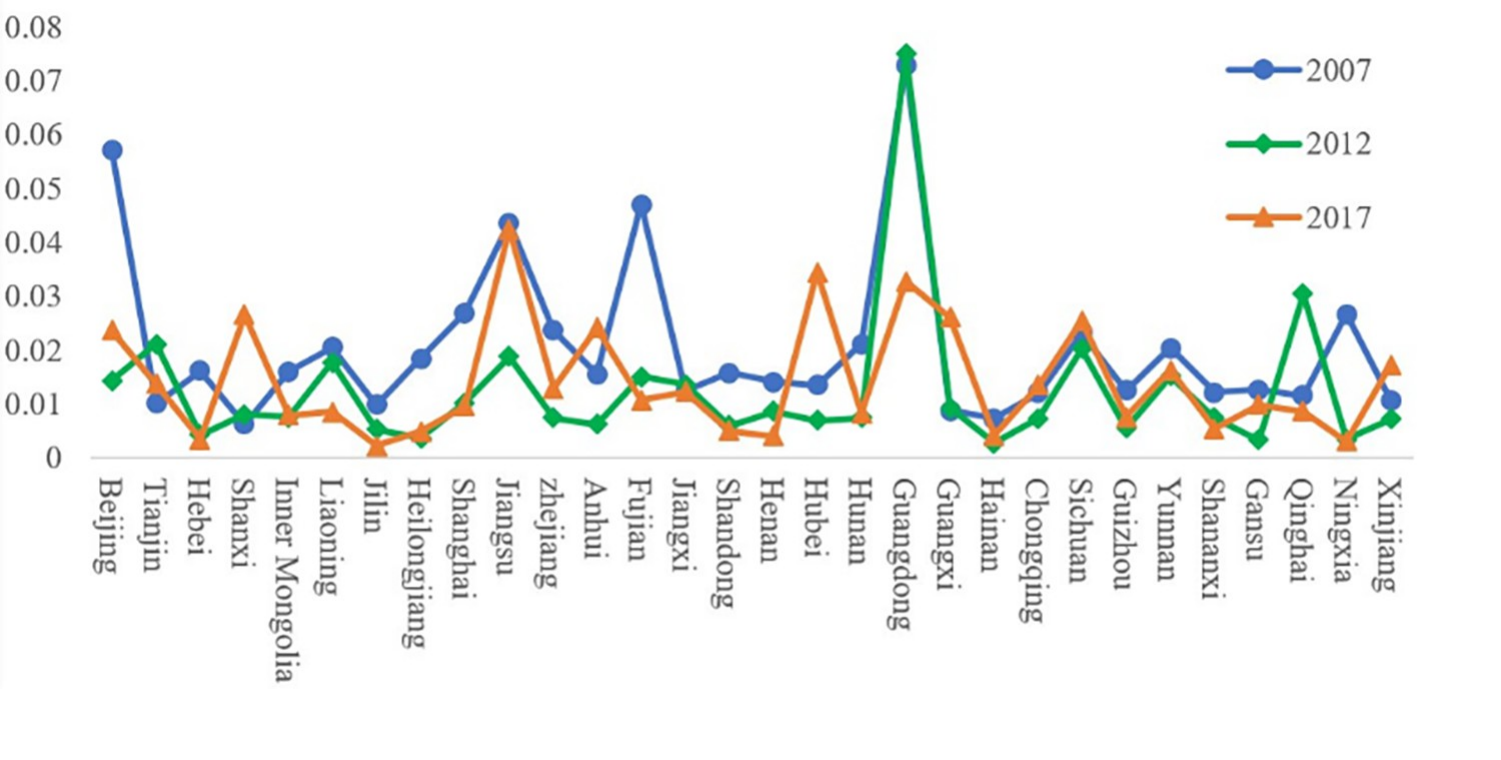
Integration level of the digital economy core industry and logistics industry in 30 provinces and municipalities in China in 2007, 2012 and 2017.

As seen from Fig 4, among the 30 provinces and municipalities in 2007, the integration degree of Guangdong Province is the highest, while that of Shanxi Province is far below the average. Overall, the integration level of southern cities is significantly higher than that of northeast China, with certain regional differences. Among southern cities, Guangdong, Beijing, Fujian and Jiangsu scored more than 0.04, showing a relatively good level of integrated development, leading by a large margin and showing good performance. This result shows that the core industry of the digital economy and logistics industry have a high level of development, and the two exhibit signs of integration. The integration level of other cities, especially Guangxi, Hainan and Shanxi, is relatively low, with scores less than 0.01. However, considering that China’s digital economy was just in the development stage in 2007, its general development level is not high, so the integration level has room for improvement.

Among the 30 provinces and municipalities in 2012, Guangdong Province had the highest degree of integration, while Hainan Province had the lowest, and the overall degree of integration was significantly lower than that in 2007. The reason can be traced back to the 2008 financial crisis. The year 2012 was the most difficult year for China’s economy since the crisis. The long-term economic malaise led to the slowdown of China’s economic development and affected the internet and logistics industry to a certain extent. In particular, Guangdong, Qinghai and Tianjin scored more than 0.02, and fusion shows a better level of development. By some distance, it shows good performance, especially in Guangdong Province. The fusion level compared with 2007 explains why the core industry of the digital economy and logistics industry development level is higher; at the same time, the two have shown stable convergence. The integration level of Qinghai Province ranks in the top three due to the government’s strong support for the logistics industry and high attention to the informatization of the logistics industry. In 2012, Qinghai Province issued several Policy Opinions on the Development of the Modern Logistics Industry in Qinghai Province, as well as relevant logistics standardisation and enterprise informatization support policies, actively promoting the application of information technology such as e-commerce, network platforms and marketing management and accelerating the process of logistics digitalization.

Thirty provinces and municipalities in 2017, more than in Guangdong Province, attained the highest level of alignment in Jiangsu Province. Jilin Province is the most general, but its overall integration level shows an obvious rise. At the same time, province alignment gradually tends toward the average, except for a few provinces, such as Hebei, Ningxia, and Jilin. The fusion level is low, and compared with an average of 0.015, there is still a large distance. Among them, Jiangsu, Hubei and Guangdong all scored more than 0.03, showing a relatively good level of integrated development, leading by a large margin and showing good performance.

On the whole, the level of integration increased year by year, and the gap between provinces and cities gradually narrowed. Second, in terms of provinces, Beijing, Jiangsu, Guangdong, Zhejiang and Sichuan have always been among the top 10 in terms of total digital economic connections in 2007 and 2017. Economically developed regions also have obvious advantages in terms of the intensity of integration. Third, in terms of the ranking of integration level, Guangdong ranked first at 0.073 in 2014, Shanxi ranked 30th at 0.006, Jiangsu ranked first at 0.042 in 2017, and Ningxia ranked 30th at 0.003. The gap between the two values is always large, which reflects the imbalance in the fusion degrees of various regions in China.

## Spatial correlation analysis of industrial integration

This paper draws on Zhao et al. [31], Ou et al. [32], Wu et al. [33], Zeng et al. [34], and other scholars’ measurement methods of regional economic connections, tourism economic connections and financial development correlation intensity. The modified gravity model is used to quantify the economic development of digital connections between China’s provincial strength based on China’s 30 provinces and municipalities in 2007, 2012 and 2017. The input–output table area is used in provinces and regions as the research unit. The regional input–output model measures 30 areas of China’s digital economy core industry and the development level of logistics integration. Based on this information, this paper introduces the gravity model to obtain the gravity relation of industrial convergence among cities and constructs the fusion network among 31 provinces.

### Building an industrial integration network

Application of the gravity model to measure urban economic ties between research has been more common. The main idea is that the economic linkages between cities are essentially their economic interactions and influences, which are most closely related to their economic level and population size. The law of economic linkages between cities is similar to gravity, the strength of economic linkages between cities is proportional to their corresponding population size and inversely proportional to the square of the distance between them.

In this paper, GDP is replaced by integration level. Based on the gravity model, the measurement formula of the gravitational relation *R*_*ij*_ of industrial integration between city *i* and city *j* can be obtained:

$$R_{ij}=\frac{\left(\sqrt{P_{i}\cdot V_{i}}\times \sqrt{P_{j}\cdot V_{j}}\right)}{d_{ij}^{2}}$$ (3)

where *P*_*i*_ and *P*_*j*_ denote the population numbers of city *i* and city *j*, respectively *V*_*i*_ and *V*_*j*_ denote the level of integration of city *i* and city *j*, respectively, and d denotes the geographical distance from city to city. Eq 3 reflects the integration gravitational link between city *i* and city *j*.

Considering that the integration gravitational effect and driving effect of two cities on each other will be influenced by a city’s integration level, which is actually different, the integration gravitational link between cities should actually be bidirectional; the integration gravitational link of city *i* to city *j* is different from that of city *j* to city *i*. Eq 4 further introduces a modified parametric equation reflecting the relative integration level of city *i* compared to that of city *j*.


$$l_{ij}=\frac{V_{i}}{\left(V_{i}+V_{j}\right)}$$ (4)

This paper combines the gravitational model with social network analysis to construct an industrial integration network. Using a series of network analysis tools, the level of integration between provinces and cities is analysed in depth; network density portrays the structural characteristics of the overall integration network; correlation reflects the robustness and vulnerability of the network; centrality reflects the importance and influence of each province and city in the network; cohesive subgroup analysis (quantitative study of small groups) identifies the existence of "subgroups" with similar structures and roles in the integration network; and core-semiedge-edge structural analysis determines the "status" of each city in the network from the perspective of the overall network.

In addition, the Netdraw program based on UCINET 6.645 software allows the visualisation of the convergence network of 30 cities. Links with a mean value of 20 or more are used for screening integration gravity. They represent valid links between city industries and take a value of 1, reflecting good industrial cooperation between cities, while links with a mean value of 20 or less represent invalid links between city industries and take a value of 0. A matrix W is generated, which more intuitively reflects the spatial evolution and spatial and temporal layout of the integration of logistics and digital industries.

### Analysis of the dynamic changes in the structure of the converged network

#### The overall structural characteristics of the network

*1*. *Network density*. Network density refers to the closeness of the connection between points in a network diagram. The degree of connectivity between nodes in the network reflects the connectivity and diffusion of the network, which in turn reflects the structural characteristics of the overall network [35]. Network density can measure the degree of interconnection between the 30 provinces and municipalities in a spatially connected network. Let the number of nodes in the network be N and the number of actual connections be M; then, the density D of the network can be expressed as

$$D=\frac{M}{N(N-1)}$$ (5)

For the fusion network constructed in this paper, the network densities in 2007, 2012 and 2017 are 0.3080, 0.4759 and 0.5586, respectively, as measured by Eq (5) and UCINET 6.645 software. This density is significantly smaller than that of the original unweighted network (in the case of not zeroing out the data for the gravitational index of industrial integration between the two provinces less than 20, taking its value in the matrix to 1 whenever there is a link between the two provinces, and the network density is 1 at this time). It shows a gradual increasing trend with time, indicating that there are relatively few provinces with an industrial integration gravitational index higher than 20 in this network at first, but with industrial development increasing annually. This pattern of growth indicates that the level of integration between the digital economy and the logistics industry is increasing annually and that the cooperation between provinces and municipalities is strengthening, with closer industrial ties and mutual enhancement of the level of integration.

*2*. *Network connectedness*. Connectedness is a measure of the robustness and vulnerability of the network. The measure of connectedness is connectedness C. Connectedness C can be measured by reachability. The range of this measure is [0, 1]. Let the number of nodes in the network be N and the number of unreachable pairs of points in the network be V. The formula for calculating the association degree C is:

$$C=1-\frac{V}{N(N-1)/2}$$ (6)

According to Eq (6), the correlation degree of the convergence network of each city is calculated. The results show that the correlation degree of this convergence network is 0.869, 0.901 and 0.933 in 2007, 2012 and 2017, respectively, which indicates that the degree of correlation of the convergence network of these 30 provinces and municipalities in China is high, the network is well connected and has a strong robustness, and there is a general level of convergence in the network spillover effect.

*3*. *Network reciprocity*. The reciprocity index is an important index in complex networks. It is used to measure the closeness of the connection between nodes and the symmetry and balance of resource access. Its formula is

$$\varphi =\frac{M_{d}}{M}$$ (7)

The network reciprocity in 2007, 2012 and 2017 is 0.696, 0.704 and 0.730, respectively, as measured by Eq (7) and UCINET software, and the reciprocity coefficient of the converged network has been above 0.65 and shows an increasing trend with time.

### Individual structural characteristics of the network

Centrality is an important indicator to measure the centrality of the whole network. In the city cluster network, the city at the centre has easier access to resources and information and has more power and a stronger influence on other cities. Network centrality can be divided into three indicators: point centrality, proximity centrality and intermediate centrality.

1. The integration and radiation ability of cities in urban clusters.

Due to the asymmetry of inputs and outputs, this paper constructs an asymmetric two-way network, so point centrality includes point-out degree and point-in degree.

Given a threshold Φ’≥20, the point-in degree is the number of connected edges that point to the city node from other cities in the network.


$$input_{i}=\sum_{j=1}^{n}I(R_{ji}⩾\Phi ),j=1,2,\cdots ,n(j\neq i)$$ (8)

A higher value of *input*_*i*_ means that the city has a higher agglomeration capacity in the convergence network.

Given a threshold Φ, the point-out degree measures whether a link exists between two cities and provinces when Φ≥20. It is determined by the number of connected edges to the city node that point to other cities throughout the network.

$$output_{i}=\sum_{j=1}^{n}I(R_{ij}⩾\Phi ),j=1,2,\cdots ,n(j\neq i)$$ (9)

where I (*R*_*ij*_≥Φ) is an indicative function to determine whether city *i* has an economic gravitational link to city *j* of at least Φ. A larger value of *output*_*i*_ means that the city has a stronger radiating power in the integration network.

As seen from Table 2, by comparing the economic radiation capacity of 30 provinces and municipalities, it is found that they can be divided into three levels, among which the high level is observed in Guangdong, Shandong, Jiangsu and Hubei, all of which are provinces and cities with a high level of their own integration and have a strong radiation capacity to their neighbouring provinces and cities; the middle level includes Henan, Sichuan, Zhejiang and Anhui, which are mostly provinces and cities in the process of economic rise and tend to increase their radiation capacity to their neighbouring provinces and cities; and the weaker level includes Hainan, Qinghai, Ningxia and Xinjiang. Weaker levels include Hainan, Qinghai, Ningxia, Xinjiang, etc. Some of these cities have yet to strengthen their own economic strength and have a weaker radiation capacity to neighbouring provinces and cities and need to accelerate their own integration level and actively link with provinces and cities with higher levels of integration in their neighbourhood.

**Table 2:** Degree centrality.

2007			2012			2017		
City	input	output	City	input	output	City	input	output
Jiangsu	21	14	Guangdong	26	10	Guangdong	26	19
Shandong	19	20	Jiangsu	25	21	Jiangsu	26	21
Guangdong	19	10	Henan	25	23	Hubei	25	20
Henan	18	19	Shandong	23	25	Sichuan	25	19
Sichuan	16	12	Sichuan	21	17	Anhui	25	19
Hunan	16	16	Hubei	20	22	Shanxi	24	15
Hubei	15	15	Hebei	20	24	Henan	24	26
Hebei	15	17	Zhejiang	20	20	Shandong	24	25
Zhejiang	14	12	Hunan	19	22	Zhejiang	24	22
Anhui	12	13	Jiangxi	17	14	Hunan	22	23

2. Analysis of the position of the city in the integration network.

Intermediate centrality, which measures the degree of control an actor has over resources, reflects mainly the role of an actor in the network as a bridge between other actors. A point is said to have a high degree of intermediate centrality if it is on a shortcut (shortest path) to many other pairs of points. The higher the intermediate centrality of city *i* is, the more that other cities need to be connected through city *i*. City *i* is also located at the heart of the integration network mediating bridges and has more control over other cities. If a city has an intermediate centrality of 0, then it cannot control and influence any other city and is at the edge of the network. This indicator is measured by calculating the ratio of the number of shortest paths of any two cities through city node *i* to the total number of shortest paths between these two cities in the city group to which it belongs, except for city *i*. The intermediate centrality *C*_*i*_ of city *i* can be expressed as

$$C_{i}=\sum_{j}^{n}\sum_{k}^{n}\frac{s_{jk}^{i}}{s_{jk}},j,k=1,2,\cdots ,n(j\neq k)$$ (10)

S_*jk*_ is the number of shortest paths between city *j* and city *k*, where S_*jk*_ is the number of paths through the city node.

The data in the table were obtained by Eq (10) and UCINET software measurement. As seen from Table 3, cities with high intermediate centrality indicators are composed of mainly provinces and cities with more developed economies in each region, Shandong and Jiangsu in East China; Hubei and Hunan in Central China, Hebei and Shanxi in North China, Shaanxi and Gansu in Northwest China, and Sichuan Province in Southwest China. These cities occupy more important positions in the integration network, with a higher status and a high degree of influence on other provinces and cities. Yunnan, Heilongjiang, Hainan, Qinghai, Ningxia and Xinjiang provinces and cities have an intermediate centrality of 0, indicating that they cannot influence other cities and are at the edge of the network.

**Table 3:** Intermediate centrality.

City	2007	City	2012	City	2017
Shandong	122.118	Shandong	66.746	Henan	44.608
Sichuan	99.055	Henan	63.418	Gansu	35.892
Guangdong	61.784	Gansu	53.000	Shandong	34.225
Henan	59.947	Guangxi	45.702	Guangdong	33.916
Hunan	55.076	Hebei	44.197	Hebei	32.880
Gansu	52.000	Jiangsu	29.059	Sichuan	32.058
Jiangsu	43.566	Shananxi	22.092	Hunan	30.163
Shananxi	43.468	Guangdong	19.815	Shananxi	17.496
Guangxi	35.461	Hunan	19.384	Jiangsu	16.039
Hebei	33.418	Hubei	16.235	Zhejiang	13.562

3. Urban centrality.

Closeness centrality portrays the ability of a point not to be controlled or influenced by other points in the network. The smaller the closeness centrality of city *i* is, the closer the city is to the core of the integrated network, and the greater the city’s ability to be free from the control of other cities. The proximity to the centre of a point can be measured by the sum of the shortcut distances between the point and the other points in the network. Denoting the sum of the shortcut distances of point *i* and point *j* by $C_{c}^{-1}(i)$, the proximity centrality of point *i* can be expressed as

$$C_{c}^{-1}(i)=\overset{n}{\underset{j=1}{\sum }}d_{ij}$$ (11)

The smaller the value of the proximity centrality of a point is, the more central that point is to the network. Notably, UCINET automatically converts the value of absolute proximity centrality into a percentage, and the higher the value is, the greater the point’s ability not to be controlled by other points in the network and the greater the centrality of the point is.

The data in the table are obtained by Eq (11) and UCINET software measurement. Table 4 shows that the mean values of in-closeness centrality and out-closeness centrality of the fusion network in 2007 were 24.846 and 24.916; the mean values of in-closeness centrality and out-closeness centrality of the fusion network in 2012 were 37.192 and 27.994, while the average values of in-closeness centrality and out-closeness centrality of the fusion network in 2017 were 40.269 and 40.590.

**Table 4:** Closeness centrality.

2007			2012			2017		
City	In	out	City	in	Out	City	in	out
Shandong	30.208	30.208	Shandong	47.541	31.868	Henan	48.333	46.774
Henan	29.897	29.897	Hebei	46.774	30.526	Hebei	48.333	43.939
Hebei	29.293	28.713	Henan	46.032	32.584	Shandong	47.541	46.774
Hunan	29.293	29.293	Hunan	43.939	30.526	Shananxi	46.774	42.029
Hunbei	28.713	29.000	Hubei	43.939	30.851	Hunan	46.032	45.313
Shanxi	28.713	25.217	Jiangsu	43.284	32.584	Zhejiang	44.615	46.774
Shananxi	28.431	27.358	Shananxi	43.284	29.293	Jiangsu	43.939	48.333
Jiangsu	28.431	30.851	Zhejiang	42.647	30.851	Sichuan	43.284	47.541
Sichuan	28.155	29.592	Anhui	42.647	29.293	Hubei	43.284	47.541
Anhui	28.155	27.619	Sichuan	40.845	31.183	Liaoning	42.647	39.726
…	…	…	…	…	…	…	…	…
Ningxia	3.333	3.333	Qinghai	3.333	26.364	Qinghai	31.522	28.155
Xinjiang	3.333	3.333	Xinjiang	3.333	3.333	Xinjiang	3.333	3.333

In 2007, the top 5 best performing provinces and cities in the fusion network in terms of closeness centrality were Shandong, Henan, Hebei, Hunan and Hubei in that order; in 2012, the top 5 best performers were Shandong, Hebei, Henan, Hunan and Hubei. From these results, the two years remained largely consistent, but this all changed five years later with Shaanxi replacing Hunan. The top 5 best performers in 2017 were, in order, Henan, Hebei, Shandong, Shaanxi and Hunan.

This result indicates that these cities and provinces have good accessibility, obvious transportation location advantages, are at the core of the trade network and are not easily controlled when making external economic connections, thus playing the role of central actors in the network. Provinces with the lowest closeness centrality are Hainan, Ningxia, Qinghai and Xinjiang, indicating that these four provinces and cities are at the edge of the network and are easily controlled by other node provinces and cities; the remaining provinces and cities have a stronger centrality in the integration network, although not as strong as Shandong and other provinces. Overall, the closeness centrality value of each province and city has a large gap, indicating that the level of coordination of industrial integration development in China is not high and needs to be improved.

4. Urban clusters and spatial distribution linkages: cohesive subcluster analysis.

The clustering analysis was carried out using CONCOR (convergent correlations iterative convergence method) in UCINET software (the maximum segmentation depth was 2 and the concentration criterion was 0.2) to further investigate whether there were "small groups" in the Chinese convergence network in 2017. The following results were obtained.

**Fig 5:**
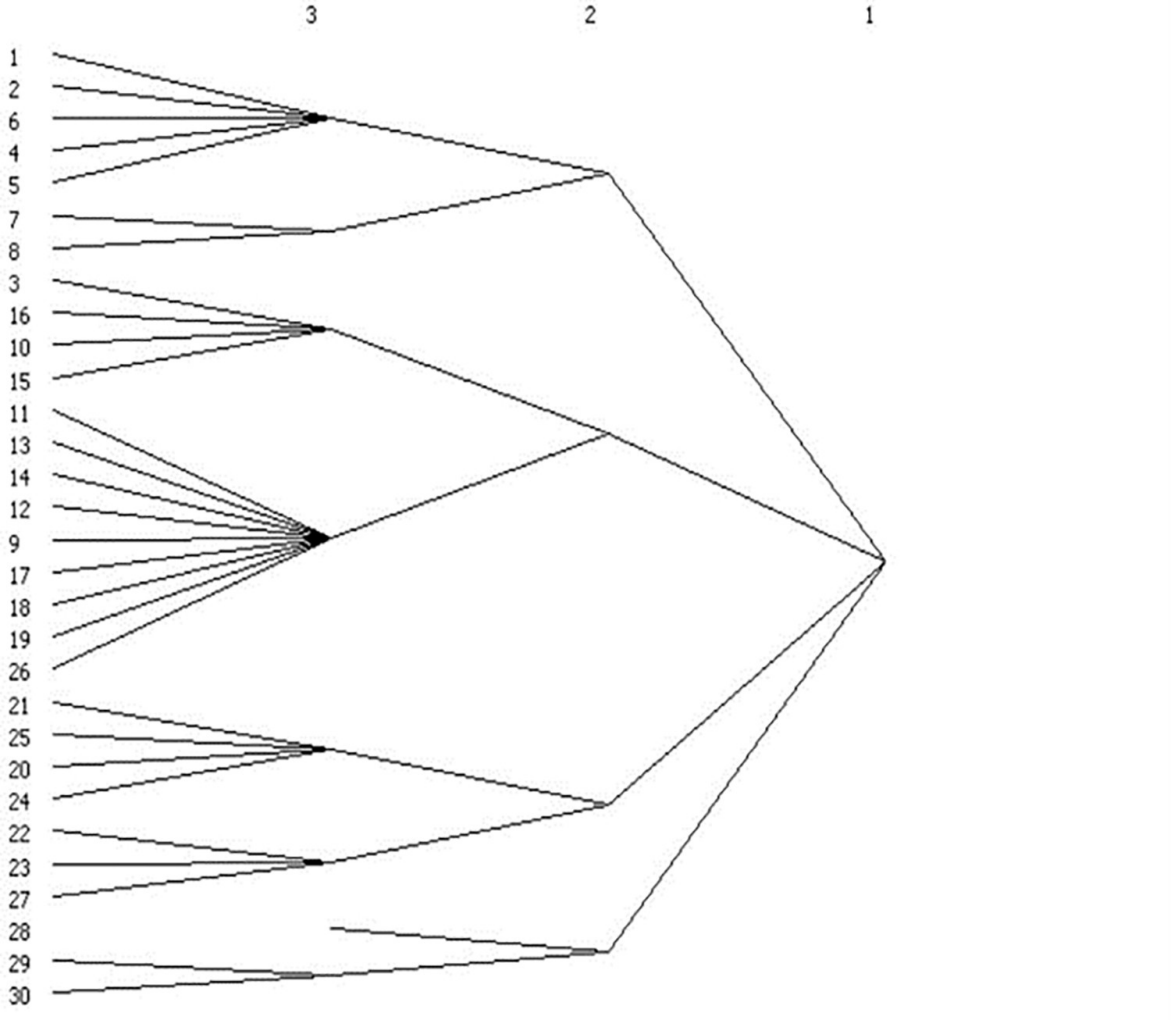
2007.

**Fig 6:**
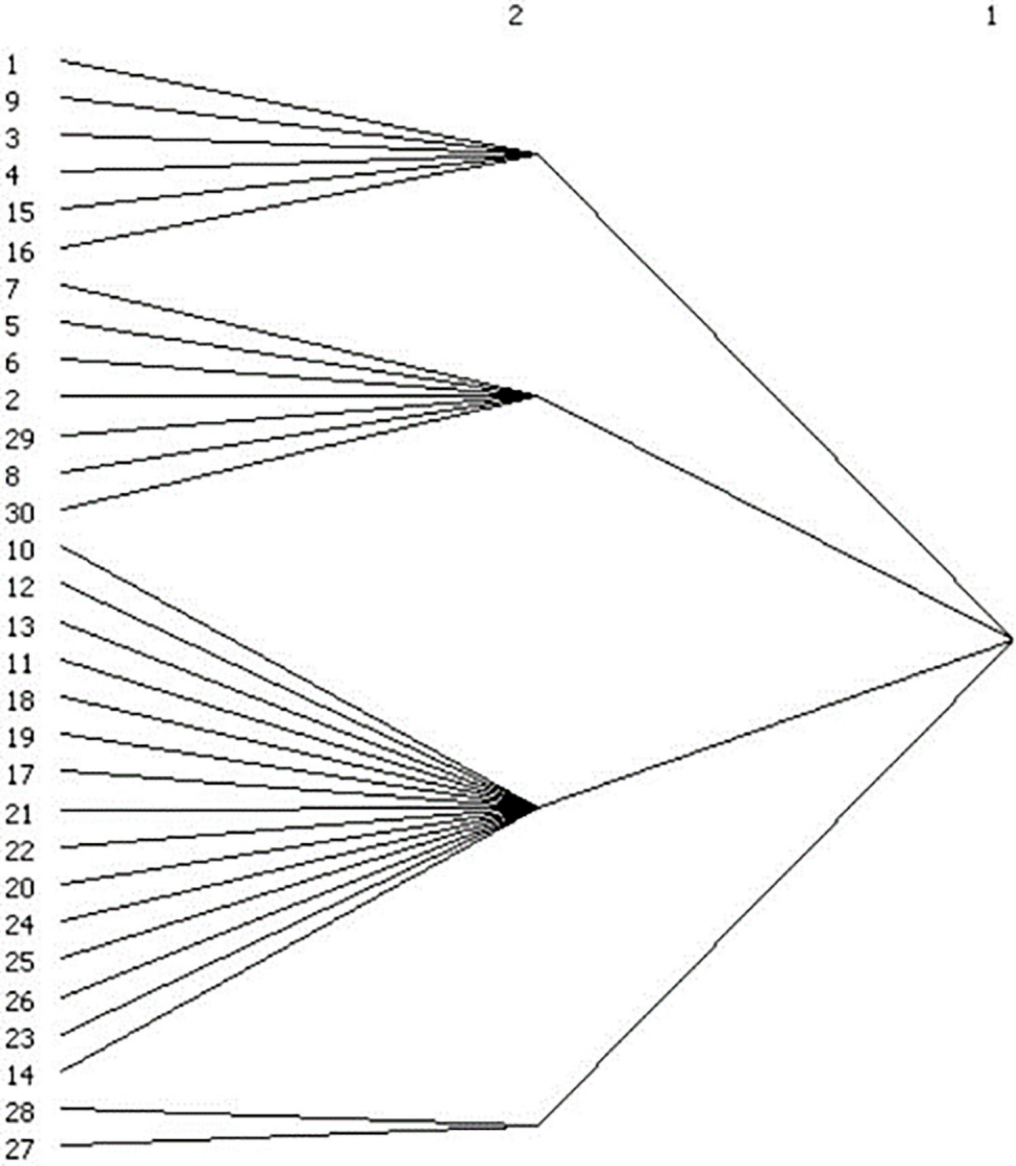
2017.

The results show that the number of subgroups decreased from eight in 2007 to four in 2017 (Figs 5 and 6), and the network changed from a three-tier to a two-tier network. The decrease in the number of groups indicates that the cities are more closely linked, while the provinces that make up each subgroup also changed, and the mode of industrial integration and development transitioned from a few cities’ industrial agglomeration to a multicity economic circle’s industrial synergy.

Specifically, in 2007, the eight cohesive subgroups on the three-tier network were as follows: the first subgroup Beijing, Tianjin, Liaoning, Shanxi, and Inner Mongolia; the second subgroup Jilin and Heilongjiang; the third subgroup Henan, Hebei, Jiangsu, and Shandong; the fourth subgroup Zhejiang, Fujian, Jiangxi, Anhui, Shanghai, Hunan, Hubei, Guangdong, and Shaanxi; the fifth subgroup Gansu, Chongqing, Guangxi, and Sichuan; the sixth subgroup Hainan, Yunnan, and Guizhou; the seventh subgroup Qinghai; and the eighth subgroup Ningxia and Xinjiang.

The division of the eight cohesive subgroups on the secondary network in 2017 was as follows: the first subgroup Beijing, Shanghai, Hebei, Henan, Shandong, and Shanxi; the second subgroup Jilin, Liaoning, Heilongjiang, Tianjin, Inner Mongolia, Ningxia, and Xinjiang; the third subgroup Jiangsu, Jiangxi, Anhui, Fujian, Zhejiang, Hunan, Hubei, Yunnan, Guizhou, Sichuan, Chongqing, Shaanxi, Guangdong, Guangxi, and Hainan; and the fourth subgroup Qinghai and Gansu.

Second, there are five groups of provinces whose members are always in the same subgroup: first, Beijing and Shanxi; second, Sichuan, Chongqing and Guangxi; third, Henan, Hebei and Shandong; fourth, Jilin and Heilongjiang; and fifth, Hainan, Yunnan and Guizhou. These provinces that are always in the same subgroup select each other more frequently based on reciprocity and thus have always maintained closer industrial integration ties and have strong group cohesion with each other. Finally, comparing the location information of the members of each cohesive subgroup reveals that the formation of each subgroup has obvious geographical characteristics, with neighbouring provinces more likely to group together to become a cohesive subgroup and develop together.

### Core-semiedge-edge structure analysis of converged networks

The core degree of 30 Chinese provinces and cities in 2007, 2012 and 2017 was calculated using UCINET 6 software, and provinces and cities with core degrees greater than 0.2 were classified as core areas, those with core degrees between 0.1 and 0.2 were classified as semiedge areas, and those with core degrees less than 0.1 were classified as edge areas. The distribution of provinces and cities in core and semiedge areas is shown in Table 5:

**Table 5:** Core degrees in 2007, 2012 and 2017.

Year	City	Core degree	City	Core degree	City	Core degree	City	Core degree	City	Core degree
2007	Beijing	0.180	Tianjin	0.085	Hebei	0.248	Shanxi	0.082	Inner Mongolia	0.078
2012		0.178		0.178		0.220		0.183		0.158
2017		0.205		0.157		0.193		0.231		0.122
2007	Liaoning	0.109	Jilin	0.034	Heilongjiang	0.034	Shanghai	0.210	Jiangsu	0.333
2012		0.178		0.039		0.039		0.146		0.279
2017		0.132		0.042		0.042		0.159		0.252
2007	Zhejiang	0.262	Anhui	0.228	Fujian	0.234	Jiangxi	0.197	Shandong	0.299
2012		0.244		0.201		0.188		0.215		0.250
2017		0.245		0.250		0.191		0.213		0.231
2007	Henan	0.302	Hubei	0.265	Hunan	0.272	Guangdong	0.294	Guangxi	0.047
2012		0.269		0.240		0.228		0.280		0.183
2017		0.231		0.250		0.226		0.252		0.194
2007	Hainan	0.004	Chongqing	0.097	Sichuan	0.235	Guizhou	0.052	Yunnan	0.033
2012		0.010		0.167		0.250		0.100		0.154
2017		0.023		0.189		0.247		0.158		0.171
2007	Shananxi	0.142	Gansu	0.029	Qinghai	0.002	Ningxia	0.000	Xinjiang	0.000
2012		0.180		0.052		0.003		0.003		0.000
2017		0.180		0.085		0.004		0.031		0.000

Using core-edge analysis, it is possible to discern the core and edge areas in the convergence network and to gain a preliminary understanding of the relationship between cities. In terms of the overall network, the core area range accounts for more than half of the total regional range, and the convergence network is beginning to develop. However, in terms of spatial distribution, the core area is basically located in the central region, although in a concentrated grouping, the western and northeastern regions show a weaker level of integration development and are distributed in the peripheral areas.

In addition, social network analysis allows visualisation of the results of the core-semifringe-fringe analysis of the trade network. The core-edge topology of the trade network is mapped by entering the weighted convergence network matrix for 2007, 2012 and 2017 into the Network program.

**Fig 7:**
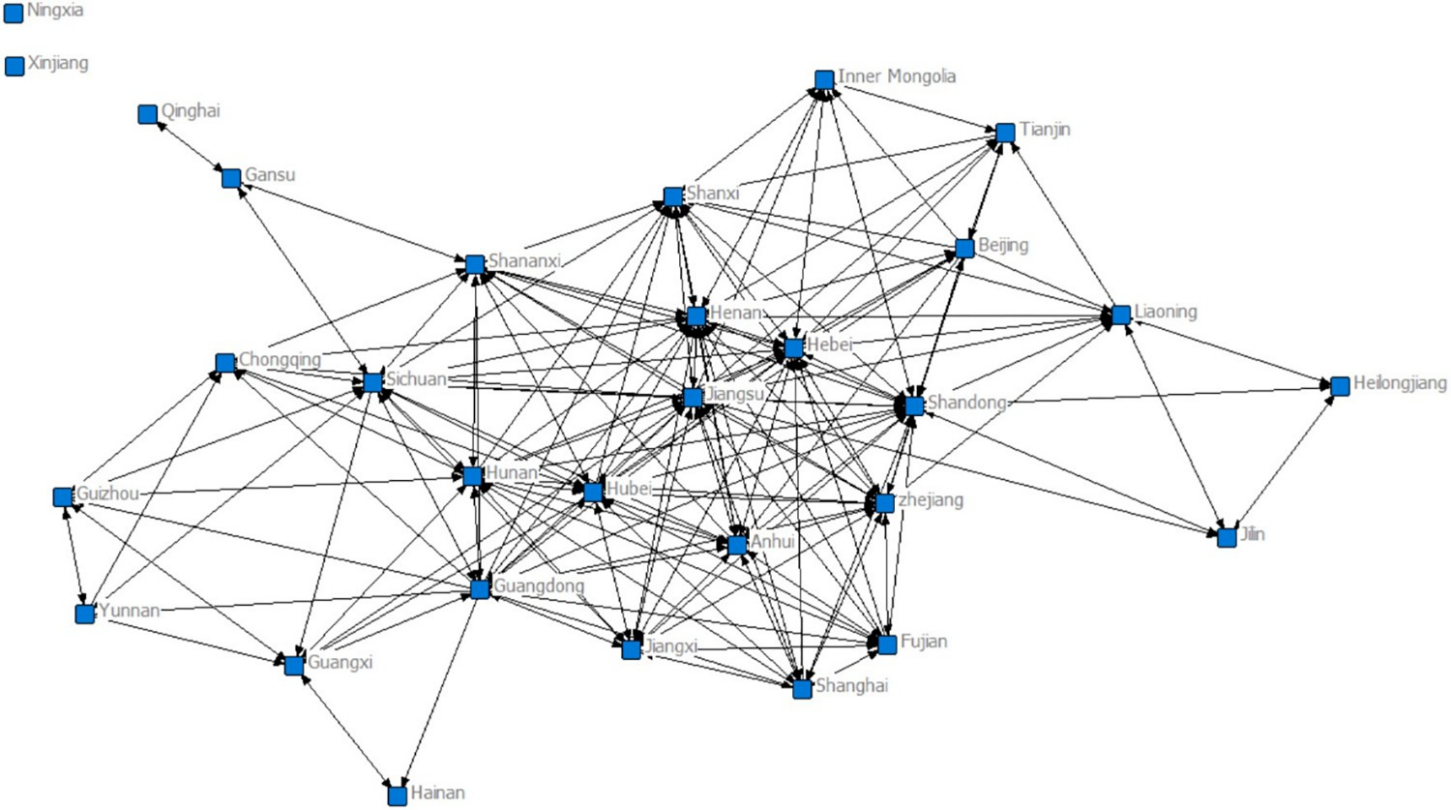
Topology of the 30-city converged network in 2007.

As shown in Fig 7, China’s convergence network was small and relatively sparsely connected in 2007. Shandong Province has connections with 20 provinces and cities, and Henan, Hebei, Hunan Guangdong and Zhejiang all have connections with more than 10 provinces and cities and are in the core of the network. Hainan, Qinghai and Gansu are at the edge of the network, with which only one or two provinces and cities have links, and Ningxia and Xinjiang do not appear in the graph because they do not have links to provinces and cities with gravitational links greater than 20.

**Fig 8:**
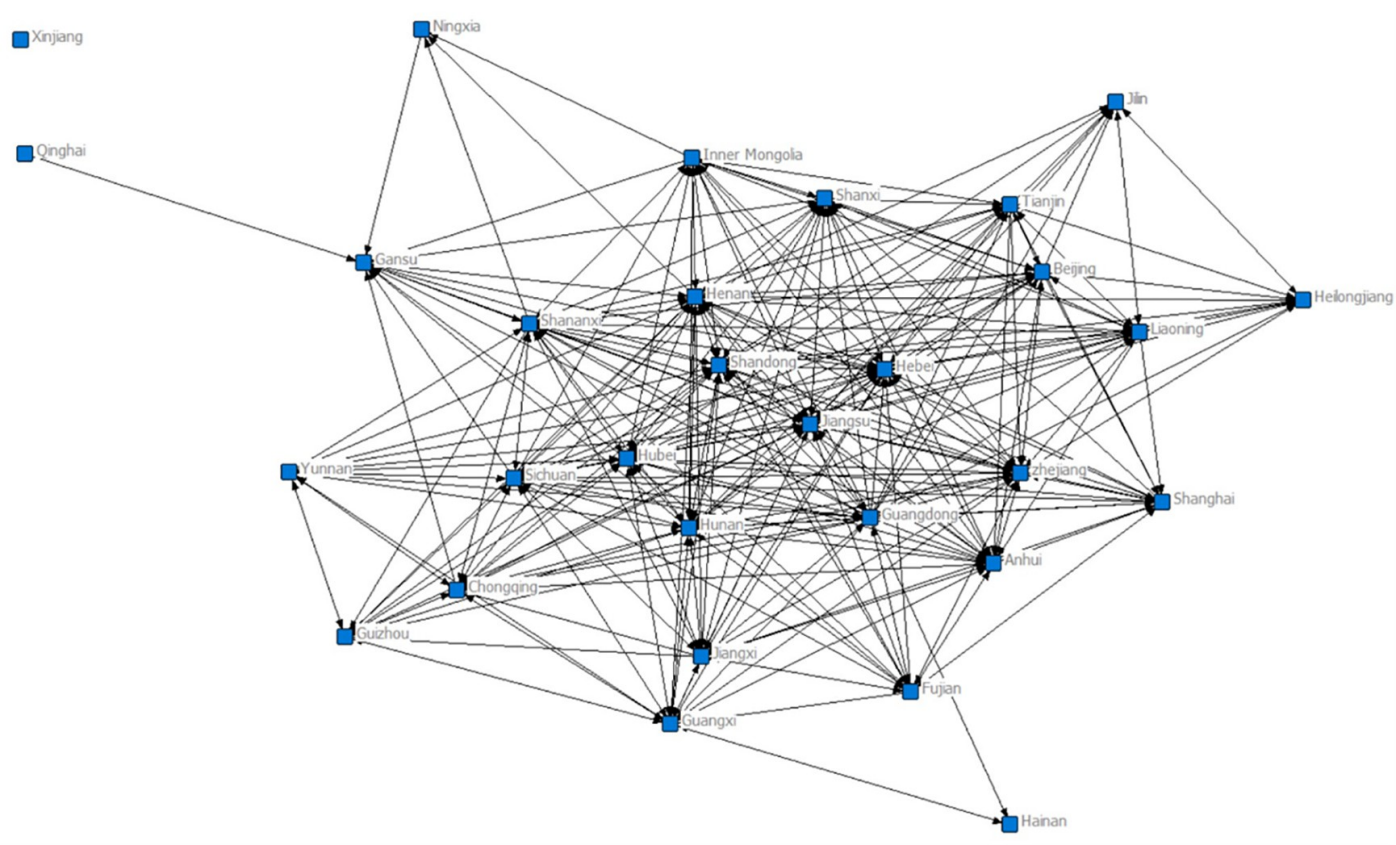
Topology of the 30-city converged network in 2012.

Connecting nodes and connecting lines all increase significantly in Fig 8, while revealing a clear edge-centre structure. The core area, represented by Shandong, Guangdong, Jiangsu and Henan provinces and cities, is gradually expanding. There are no provincial-city associations with gravitational links greater than 20 in Qinghai and Xinjiang. There is no change in the number of cities, but the number of association paths has increased, the overall network size has expanded significantly compared to 2007, and the stability of the network structure has been enhanced.

**Fig 9:**
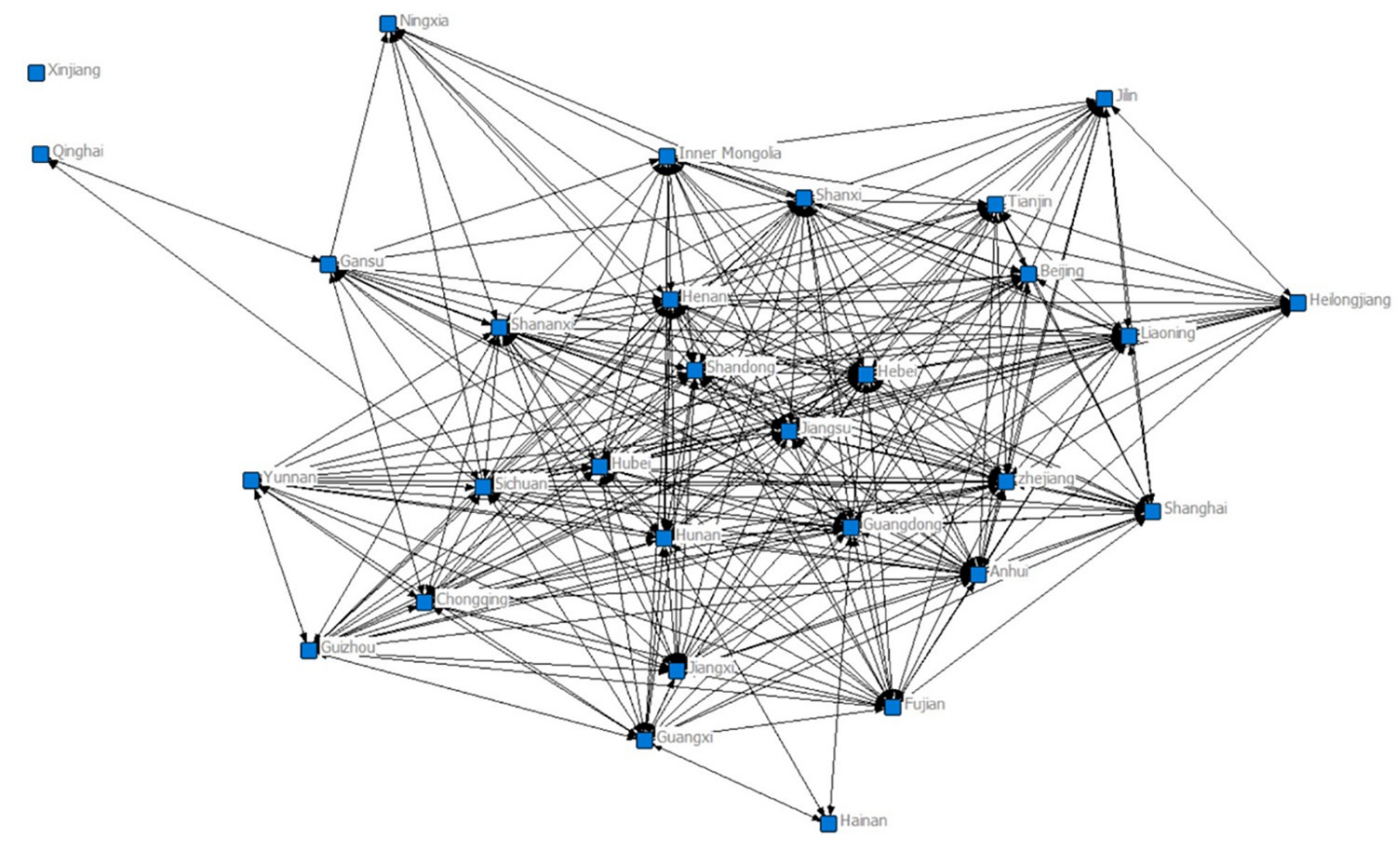
Topology of the 30-city converged network in 2017.

Fig 9 shows that the connected nodes and links continue to increase, and the network shows a trend of decentralisation. The core zone, represented by Guangdong, Hebei and Hubei, occupies more than half of the area, while Sichuan and Shanxi have entered the core zone. The number of provinces and cities in the semiperipheral zone has decreased, and provinces and cities in the peripheral zone, such as Qinghai and Ningxia, have increased their connectivity with other provinces and cities, but there are still no interprovincial links with gravitational links greater than 20 in Xinjiang. There is no city in the network that has absolute dominance over other cities, the node cities interact and influence each other. The network shows a synergistic development trend, gradually evolving towards a multiwin pattern of balanced and common development.

From the three trade network topology diagrams, we can see that (1) the nodes in the diagram show an obvious core-edge structure, with the core provinces and cities located in the core circle of the trade diagram, the semiedge provinces and cities in the middle circle, and the edge provinces and cities distributed in the outer circle. (2) The size of the nodes in the diagram represents the importance of the province and city in the trade network; the larger the node is, the more important the province and city are. In addition, the size of the node is consistent with the core degree of the node; that is, the greater the centrality of the individual in the integration network, the greater its core degree in the network. The larger a node is, the more central it is in the central region, and the smaller a node is, the more marginal its location is. (3) The number of links in the diagram is directly proportional to the industrial links between provinces and cities. Nodes in central and eastern provinces and cities have a larger rectangle size than nodes in other regions, and their nodes have more links, indicating that the central and eastern regions of China are more central than other regions and are in a more central position in the network. The closeness of the fusion network links between southern provinces is also stronger than that of northern provinces. (4) Comparing the sparseness of the overall network linkages in Figs 7–9, it can be seen that Fig 9 has more and denser linkages, which indicates that the industrial integration linkages between provinces in China are stronger in 2017 than in 2007. In addition, marginal provinces such as Hainan, Qinghai and Ningxia, which were less connected in 2007, all saw an increase in the number of connected edges in 2017, indicating that these provinces also actively integrated into the industrial integration network over time and are gradually playing a role in the network.

## Conclusions

This paper uses input-output data provided by the National Bureau of Statistics from 2007–2017 for the whole country and 30 provinces, cities and autonomous regions to construct a directionally weighted integration network with 30 provinces and cities as nodes and the integration gravitational force between provinces and cities as edges, and uses the ranked hierarchical network method to systematically analyse the degree of industrial integration and spatial association among 30 provinces and cities in China, and to explore their main association factors. The main conclusions show the following: Firstly, the overall integration between the core industries of China’s digital economy and the logistics industry is in a good trend, with the degree and growth rate of integration increasing as the digital economy develops year by year, but the integration level of some marginal less-developed provinces is not satisfactory. Secondly, there are asymmetry and unevenness in interprovincial industrial integration links. Overall, China’s industrial integration links manifest mainly as economic radiation effects. In terms of spatial distribution, the spatial distribution of the integration network shows an evolutionary pattern of radiation and diffusion from the central region to the western and north-eastern regions. In addition, there is a large north–south difference in the level of industrial integration, with southern provinces mostly showing the radiation effect and northern provinces mostly showing the agglomeration effect. Finally, the overall network shows a clear edge-core structure, with provinces at the core of the network show strong cohesion, while those at the edges of the network show adsorption ability around the core. Additionally, the analysis of the cohesive subgroups of the network shows that the cohesive subgroups are gradually stabilising, the neighbouring provinces in the fusion network are more likely to group together as the same cohesive subgroup and develop together.

## Policy implications

In summary, it can obtain the following policy implications from this paper.

Firstly, accelerate the construction of new infrastructure in each province and city, encourage digital technology innovation and development, and provide digital technology support for the digitalization of the logistics industry. While building logistics infrastructures such as roads, ports, airports and stations, provinces and cities should coordinate sensor systems such as 5G networks, the BeiDou Navigation Satellite System (BDS), other region-wide layouts and a new type of infrastructure about integrative aerospace to provide environmental support for the transmission of circulation data, drive the development of physical logistics networks such as production logistics, transport logistics, cold chain logistics, bonded logistics and trade logistics, and internet logistics networks such as freight forwarding, cross-border e-commerce and terminal distribution, and to radiate drives the construction of support areas such as equipment, finance and parks within the logistics industry. At the same time, local governments at all levels should provide policy support to create a good external environment for the digital transformation of logistics enterprises. Support for investment in logistics infrastructure and information facilities should be increased, with support the construction of logistics information platforms for logistics enterprises actively, and encourage logistics enterprises to adopt a new generation of digital technology.

Secondly, to solve the problem of the uneven development of the integration of the digital economy and the logistics industry between the north and the south, we should promote industrial integration by region, fully exploit the agglomeration effect of integration resources in the northern provinces and the radiation effect in the southern provinces, optimise the market allocation of resource elements, and realise mutual benefits and win–win situations in the north and the south through the cross-regional integration of digital resources and logistics and transportation. To seize the opportunity result from the fact that the degree of integration is increasing annually, grasping the regular characteristics of the integration trend evolving from the central region to the surrounding area and further spreading to the western region, unifying the spatial layout of the interprovincial integration network and allowing marginal areas to participate in integration. At the same time, given the different positions of different provinces in the industrial integration network, a differentiated development strategy will be implemented to leverage the driving role of the core provinces of the network, and to pay attention to the coordination of the construction of new infrastructure between regions, thus narrowing the infrastructure gap between east and west and urban and rural areas and realising the efficient sharing of circulation data and information.

Finally, following the development trend of using the centre to drive the periphery, attaching importance to the radiating role of the central cities of each subcluster in industrial cooperation. Taking advantage of the developed provinces’ strengths in digital infrastructure construction, digital human resource reserves, digital technology development levels, logistics technology and equipment, the mobility of the core industries of the digital economy and the logistics industry should be exploited, and the integration of resources should be promoted. The links between economic circles should be strengthened, economic cooperation among neighbouring provinces should be strengthened, and the advantages of core hub cities, regional hub cities and port hub cities within each economic circle should be leveraged to link the construction of digital logistics in surrounding regions and to encourage the continuous spread of integration networks to the periphery. Provinces and cities within the subgroup should be encouraged to cooperate and exchange in terms of capital, technology, talent and other resources to jointly build an interactive platform for the integration and development of the logistics industry and the digital economy, encourage the sharing of data resources in some industries, play an active role in the communication and linkage of interprovincial platforms, disrupt information silos and technical bottlenecks, providing help to other provinces and cities transformation.

## Research limitations and future prospects

The current development of digital economy has become the wave of the times, and the digitalization of logistics has been an inevitable trend for the logistics industry to achieve high-quality development. In this regard, this paper systematically study the existing research results on industrial integration, combine the current situation of the development of China’s digital economy and logistics industry, take into account the boundary nature of the industry, combine the reliability and availability of data, use the input-output method and SNA, for the first time to China digital measure the development level of economy and logistics industry, and characteristics are analyzed based on space and time, It enriches the blank of relevant research and provides certain reference value for follow-up research.

Whlie, due to the limitation of time, energy and space, this paper only starts with the static input-output method and calculates the integration level of the core industry and logistics industry of the digital economy in combination with SNA. However, due to the limitation of the availability and reliability of data, only the discontinuous five years from 2007 to 2017 are selected as research data, lacking the latest data in recent years. Due to the lack of detailed research on the measurement of the industrial integration level between China’s digital economy and logistics industry, there is still a gap between the calculated integration results and the actual situation.

Future research can use dynamic input-output model to supplement the input-output table data, further improve the reliability of this measurement method, and to a certain extent, more complete and scientific research on the industrial integration measurement of China’s digital economy and logistics industry. At the same time, due to the availability of data, this paper only considers the input-output method and SNA, but future research can also combine measurement methods such as measurement model and indicator system to expand the measurement method of industrial integration level.
